# Design and Hysteresis Compensation of Novel Resistive Angle Sensor Based on Rotary Potentiometer

**DOI:** 10.3390/s25134077

**Published:** 2025-06-30

**Authors:** Ruiqi Liu, Min Li, Jiahong Zhang, Zhengguo Han

**Affiliations:** 1 School of Integrated Circuits, Nanjing University of Information Science and Technology, Nanjing 210044, China; ruiqi_liu@nuist.edu.cn; 2 Jiangsu Collaborative Innovation Center on Atmospheric Environment and Equipment Technology, Nanjing 210044, China; 3 School of Automation, Nanjing University of Information Science and Technology, Nanjing 210044, China; hanzhengguo@nuist.edu.cn

**Keywords:** angle sensing, rotary potentiometer, industrial automation, adaptive linear neuron, hysteresis error compensation

## Abstract

Resistive angle sensors are widely used due to their simple signal conditioning circuits and high cost-effectiveness. This paper presents a resistive angle sensor based on a rotary potentiometer, designed to offer a measurement range of 180° for low-cost angle measurement in industrial automation and electromagnetic interference (EMI)-sensitive applications. The sensor features a specially designed signal conditioning circuit and mechanical housing. Experimental results show that it exhibits excellent linearity and temperature stability over a wide temperature range of −20 °C to 60 °C, with a zero-temperature drift of approximately 0.004°/°C. For the nonlinearity and hysteresis caused by unavoidable friction and manufacturing tolerances between the transmission mechanism and rotary potentiometer, an adaptive linear neuron (ADALINE) technique based on the α-least mean square (α-LMS) algorithm was implemented for software compensation. The results show that the percentage nonlinearity error was reduced from the original 4.413% to 0.182%, and the percentage hysteresis error was decreased from the original 4.061% to 0.404%. The research results of this paper offer valuable insight for high-precision resistive angle sensors.

## 1. Introduction

With the development of industrial automation, robotics, and automotive technology, there is a growing need for angle sensors that are accurate, low-power, reliable, insensitive to environmental conditions, and maintenance-free [1]. Significant global progress has been made in both non-contact and contact angular sensing research over the past two decades. However, challenges remain in achieving cost efficiency, environmental robustness, and immunity to electromagnetic and physical interference.

Non-contact angle sensors primarily operate based on capacitive, inductive, magnetoresistive, and optical principles. Capacitive sensors measure angular displacement through variations in the overlapping area between concentric circular electrode arrays [2,3]. Goto et al. developed a bending angle sensor based on double-layer capacitance for detecting joint angles during cycling motion [4]. Inductive sensors determine angular position through variations in coil self-inductance or mutual inductance between multiple coils. For instance, Wu et al. proposed an inductive angle sensor based on the principle of electromagnetic induction, composed of a magnetic core, a primary coil, and a secondary coil. This sensor achieves ±40″ accuracy across 0–360° and is suitable for angular displacement measurement in large hollow rotating machinery [5]. Most of the reported magnetoresistive angle sensors consist of a magnetic orientation sensor integrated on a chip and a rotating magnet mounted on a shaft [1]. Chen et al. introduced a cross-orthogonal architecture based on Hall sensors to locate the angle. The angle measurement method enables 0° positioning and 360° planar angle measurement with an overall system error of ±1.263° [6]. Optical angle sensors are widely adopted for their ultra-high resolution [7], and a recent study [8] proposed a polymer optical fiber (POF) sensor system for multi-planar angular estimation in structural health monitoring and robotic applications. Errors as low as 1.15% in the best case and 4.30% in the worst case were obtained on the tests with multi-plane movements.

Although the above methods exhibit low power consumption and high resolution, their performance is considerably constrained in EMI-sensitive environments. For instance, in industrial automation scenarios, angle sensors are subjected to complex EMI generated by large-scale motors and frequency converters. Similarly, in outdoor applications near radar installations or signal towers, sensors face additional challenges from electromagnetic radiation. Under such conditions, capacitive angle sensors are susceptible to parasitic capacitance and magnetoresistive-type sensors exhibit accuracy degradation due to discrepancies between the detected and actual magnetic field [1]. Optical angle sensors are too costly and unsuitable for contaminated industrial environments. In contrast, the contact angle sensors (resistive) have great advantages by virtue of their low integration complexity, insensitivity to environmental interference (e.g., dust/oil), extremely low cost, and high flexibility [9,10]. Recent studies primarily apply resistive angle sensors to wearable technology and robotic joints [11,12,13,14], with limited industrial control applications reported. These sensors typically have an angular measuring range of less than 180° and an accuracy of ranging from 0.5° to 3°. This does not mean that the problem of resistive angle sensors in industrial and EMI environments has been solved. The direction of how resistive angle sensors can improve accuracy and environmental adaptability while retaining the low-cost advantage and overcoming non-ideal factors such as hysteresis in the above applications has not been fully explored in the existing literature. Therefore, it is necessary to conduct a targeted study of contact-based solutions.

The design of resistive angle sensors centers on potentiometers, which convert angular displacement to voltage via a movable contact (wiper). These sensors are widely used due to their simple signal conditioning circuit and high cost-effectiveness [15]. Moreover, the fundamental potentiometric principle makes their performance in EMI-sensitive applications virtually unaffected by electromagnetic fields. However, resistive angle sensors exhibit inherent limitations including mechanical wear and material degradation of their movable contacts or wipers over prolonged operational cycles [16]. In addition, unavoidable manufacturing tolerances between the transmission mechanism and potentiometer can cause nonlinearity and hysteresis errors, reducing accuracy.

Sensor hysteresis refers to the irreversible difference in the sensor output response due to the different historical change paths of the input signal during dynamic measurement. This phenomenon is a key factor affecting the long-term stability and measurement repeatability of high-precision sensors. Its essence stems from the non-ideal properties of the material or structure inside the sensor, such as the viscoelasticity of the material, residual stress relaxation, and physical gaps in the structure. Specifically, this is manifested by the systematic offset of the output signal when the sensor reaches the target point from the forward and reverse paths with the same input [17]. At present, hysteresis compensation research mainly targets piezoelectric and humidity sensors, as they have significant hysteresis errors. Compensation methods involve empirical models, support vector machines (SVMs), and neural networks [18,19,20]. However, there is relatively little research on the hysteresis characteristics of resistive angle sensors. Hollingshead et al. investigated the hysteresis characteristics of resistive bending sensors [21], but they neither identified causes nor proposed compensation methods. Islam et al. proposed a software technique based on an artificial neural network (ANN) to compensate for nonlinearity and hysteresis errors in nanostructured, porous silicon relative humidity sensors [22]. This method makes the output and input of the sensor approximately linearly related, and the output characteristic curves of the forward and reverse strokes tend to coincide. It can be used to solve the hysteresis problem of the resistive angle sensor. Moreover, it is simple to implement and has strong portability.

To address the growing demand for cost-effective angular measurement in industrial automation and EMI-sensitive environments, this paper presents a novel resistive angle sensor based on a rotary potentiometer. It is incorporated with a mechanical housing which features a manual calibration function and a signal conditioning circuit based on the principle of analog-to-digital conversion (ADC) to measure the rotation angle of devices in industrial control fields. To enhance accuracy, an ANN based on the α-LMS algorithm was used to perform software compensation and simulation studies on the nonlinearities and hysteresis phenomena exhibited by the sensors during the experimental process.

## 2. Methods

### 2.1. System Architecture

**Figure 1:**
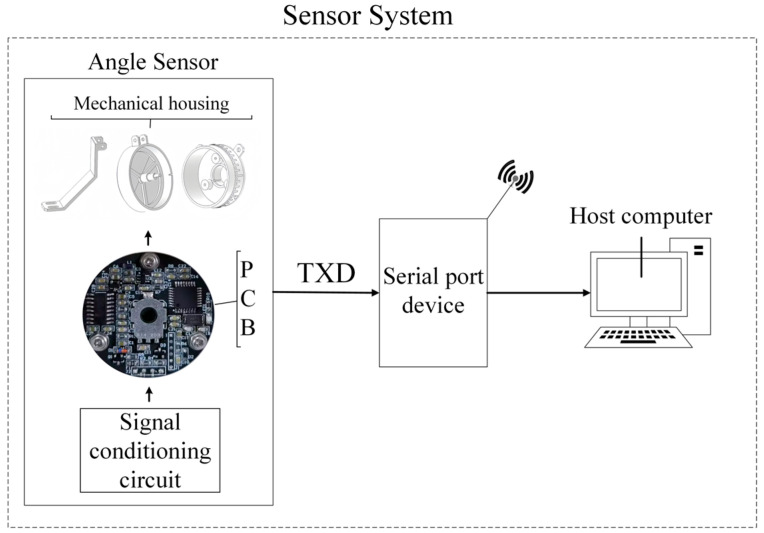
Overall system architecture.

The proposed angle measurement system consists of three parts: the angle sensor, a serial device, and a host computer. Specifically, the angle sensor includes a signal conditioning circuit, a PCB, and a mechanical housing. The signal conditioning circuit is integrated into the PCB, which is secured to the mechanical housing by three screws, responsible for collecting angle data and transmitting it to the serial device. Upon receiving the data, the serial device sends it to the host computer via the recommended standard 232 (RS-232) [23] or universal serial bus to universal asynchronous receiver/transmitter (USB-UART) protocols. Finally, the user reads the sensor data from the host computer. The overall system architecture is shown in Figure 1.

### 2.2. Principle of Operation

**Figure 2:**
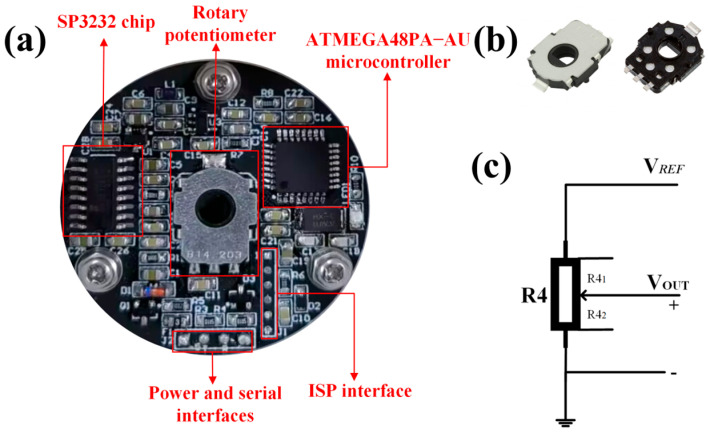
(**a**) The photograph of the core circuit board; (**b**) the rotary potentiometer; (**c**) the topological structure of the moving contact equivalent circuit.

The core circuit of the system is shown in Figure 2a, which mainly consists of a rotary potentiometer, a microcontroller, a SP3232 chip, an in-system programming (ISP) interface, a power and serial interface, and a signal conditioning circuit. The microcontroller is software-programmed and flashed to the core board via an ISP burner and an ISP interface. The system measures relative angular displacement in industrial control systems by acquiring the sensor’s linear voltage output. The principle of operation is based on the linear relationship between the angular displacement of the rotary potentiometer and the change in its resistance value: when the rotary shaft undergoes mechanical displacement, the potentiometer’s resistance changes, causing the sensor to generate a linear voltage output corresponding to the rotation angle. The voltage output increases linearly with the rotation angle [15]. The rotary potentiometer used is the EVWAE4001B14 from PANASONIC, with an electrical rotation angle range of 0–343°, capable of operating 1 million cycles. Its overall maximum size tolerance is 0.6 mm, and the maximum size tolerance of the center hole is 0.2 mm, as shown in Figure 2b. The topological structure of the device’s moving contact equivalent circuit is shown in Figure 2c. According to the standard voltage divider principle, the output voltage *V_out_* can be expressed as follows:

(1) $$V_{\mathrm{out}}=\left(\frac{R4_{1}}{R4_{1}+R4_{2}}\right)V_{REF}$$

where *R*4_1_ is the upper half resistance of the rotary potentiometer, *R*4_2_ is the lower half resistance of the rotary potentiometer, and *V_REF_* is the reference voltage of the rotary potentiometer.

### 2.3. Microcontroller and Circuit

To balance measurement accuracy and cost-effectiveness, this system employs the ATMEGA48PA-AU microcontroller as the main controller with a built-in 10-bit ADC. Its supply and reference voltages are both 3.3 V, and it is integrated with the signal conditioning circuit on the PCB. A passive crystal oscillator provides it with a system clock of 11.0592 MHz. The minimum resolvable voltage of its ADC, defined as the least significant bit (LSB), can be expressed as follows:

(2) $$V_{LSB}=\frac{V_{REF}}{2n}=\frac{3.3V}{2^{10}}=\frac{3.3V}{1024}\approx 0.003V$$

where *V_LSB_* is the least significant bit of this microcontroller, *V_REF_* is the reference voltage of this microcontroller (3.3 V), and *n* is the number of bits of the ADC.

Therefore, the minimum resolvable angle (accuracy) of the sensor can be expressed as follows:

(3) $$\theta_{LSB}=V_{LSB}\times \frac{\theta_{R}}{V_{PREF}}\approx 0.003V\times \frac{343{}^{\circ}}{3.3V}\approx 0.31{}^{\circ}$$

where *θ_LSB_* is the minimum resolvable angle (accuracy) of the sensor, *θ_R_* is the electrical rotation angle of the potentiometer, and *V_PREF_* is the reference voltage of the potentiometer.

According to the microcontroller chip datasheet recommendations, the ADC clock frequency should be maintained within the range of 50 kHz to 200 kHz to achieve the maximum conversion accuracy. Therefore, the sensor system is configured with an ADC clock frequency of 172.8 kHz, corresponding to a sampling rate of approximately 12.5 kilo samples per second (kSPS). The data transfer rate is approximately 200 kbit/s, meeting the timing requirements of most commercial servo or motion control systems with medium-speed and low-speed response needs. Additionally, this sensor has multiple communication interfaces, facilitating integration with other systems and data interaction. It should be noted that replacing the microcontroller chip with a higher-performance one can achieve a higher sampling rate and enhanced data transfer capacity, but this will increase system cost.

**Figure 3:**
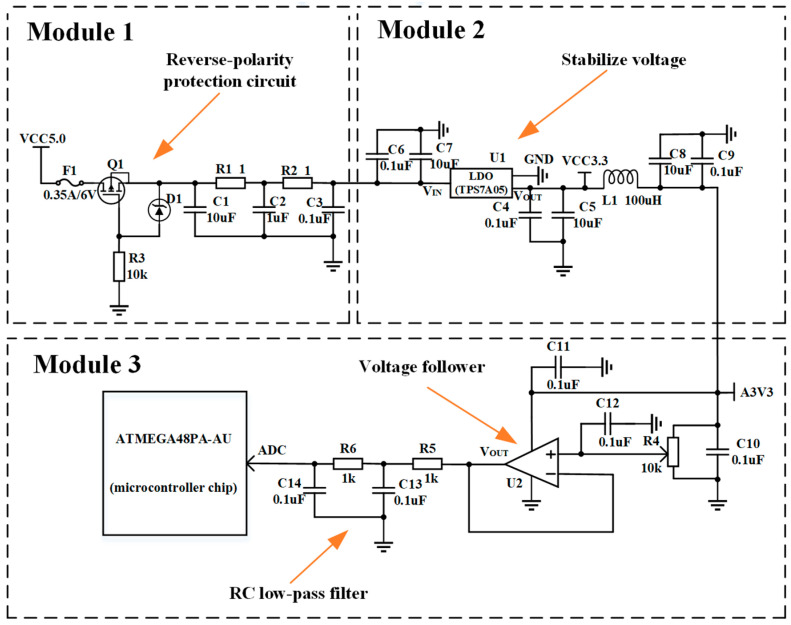
The circuit diagram of the resistive angle sensor.

The circuit design of the sensor is the system’s core. Excellent hardware circuit design reduces the production cost while ensuring the measurement accuracy and also lays the foundation for the hysteresis error compensation. The specific circuit diagram is shown in Figure 3. The circuit can be divided into three modules, which collectively achieve sensor voltage acquisition, filtering, voltage conversion and regulation, protection, and communication with the host computer. In Module 1, the fuse F1, metal–oxide–semiconductor field-effect transistor (MOSFET) Q1, and Zener diode D1 form a reverse-polarity protection circuit to prevent incorrect polarity connection at the input terminals. Decoupling capacitors C1, C2, and C3, along with resistors R1 and R2, are employed for conducting power supply filtering to ensure a stable 5 V DC output voltage. Module 2 incorporates a low-dropout regulator (LDO, TPS7A05) to convert the 5 V DC voltage into a stable 3.3 V DC voltage, which powers the rotary potentiometer, the operational amplifier U2, and the microcontroller. The output voltage temperature coefficient of this LDO is ±50 ppm/°C, thus improving the temperature stability of voltages. Its extremely low output voltage noise (7.8 μV RMS) and high PSRR (55 dB at 1 Mhz) at high frequencies suppress electromagnetic noise. Decoupling capacitors C4, C5, C6, C7, C8, and C9 and inductor L1 are used for filtering. Module 3, as previously mentioned, includes the wiper circuit. Its output voltage is routed through a buffering circuit implemented with the MCP6V91T-E/OT from Microchip, then transmitted to the microcontroller’s built-in ADC pin for real-time sampling. Finally, the microcontroller processes the signal and uploads the data to the host computer via a serial device. The MCP6V91T-E/OT functions as a voltage follower, isolating the resistive voltage divider network formed by the rotary potentiometer from the subsequent circuit. This eliminates the impact of load effects on the voltage division ratio while simultaneously suppressing the output voltage noise from the rotary potentiometer. By providing a stable signal source for the microcontroller’s built-in ADC, it enhances measurement accuracy. The input offset voltage temperature drift of the operational amplifier is extremely low, i.e., only ±17 nV/°C. Moreover, its internal design includes an EMI suppression circuit, and its EMI suppression ratio at high frequency (1.8 Ghz) reaches 93 dB, which further suppresses the electromagnetic noise. Capacitors C13 and C14, in conjunction with resistors R5 and R6, form a second-order passive RC low-pass filter, with a cutoff frequency of approximately 1591.5 Hz. It can effectively filter out high-frequency interference, enhancing signal stability. Decoupling capacitors C10, C11, and C12 are used for filtering.

One of the significant cost advantages of the angle sensor proposed in this study lies in its simple manufacturing process. Signal processing is accomplished using low-cost electronic components on the PCB and the built-in ADC of the microcontroller, eliminating the need for complex machining procedures. In contrast, conventional resistive angle sensors used in engineering applications require external ADCs and complex embedded circuits [9,24]. Although non-contact angle sensors can achieve a measurement accuracy ranging from 0.01° to 0.1° by relying on precision grating materials, precisely mounted permanent magnets, and costly optoelectronic components [25,26,27], the cost-effectiveness of this resistive angle sensor has a great advantage in some industrial automation and EMI scenarios that are cost-sensitive and where a 0.5° accuracy is sufficient.

### 2.4. Mechanical Housing of Sensor

**Figure 4:**
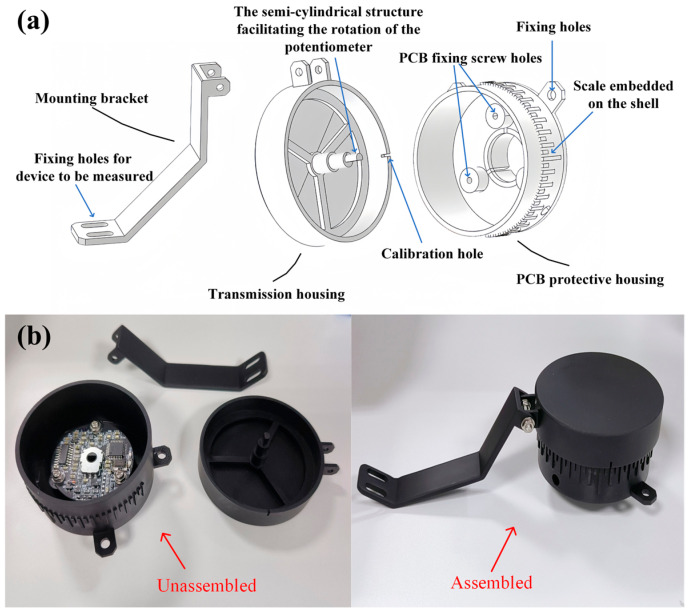
(**a**) The design drawing of the mechanical housing; (**b**) the photograph of the mechanical housing.

The mechanical housing of the sensor has been specially designed, which contains the mounting bracket, transmission housing, and PCB protective housing. Materials such as metal, nylon, and resin can be selected according to actual application needs. The mounting bracket, fixed on the device under test via screw holes and screws, ensures a secure connection. The semi-cylindrical structure in the transmission housing is embedded into the semi-circular hole in the center of the rotating potentiometer. The rotation of the industrial equipment to be measured drives the transmission housing, converting mechanical motion into potentiometer angular displacement via the semi-cylindrical structure. The PCB is fixed to the PCB protective housing with three screws positioned at mutual 120° angles. Moreover, the calibration hole on the transmission housing and the scale inlaid in the protective housing are utilized for angle calibration and demarcation. With the scale marked every 5°, the current angle can be determined once their centers are aligned. This unique design simplifies the manual angle calibration process. The overall design of the mechanical housing is shown in Figure 4a. Figure 4b presents the photograph of the sensor’s mechanical housing.

## 3. Experiments

### 3.1. Consistency Testing

**Figure 5:**
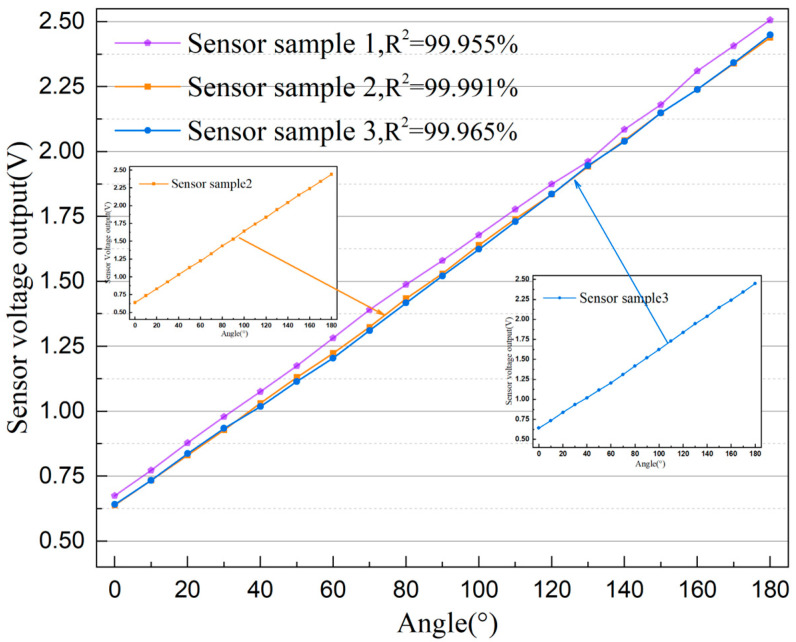
The output characteristic curves of the three tested sensor samples.

To evaluate the consistency of angle measurement performance for the designed same-batch sensors, this study conducted a multi-sample consistency test at room temperature. In the test, this angle sensor was mounted, and the transmission housing was manually rotated to the 0° position. Then, the transmission housing was slowly pushed toward 180°, causing the rotary potentiometer to generate the corresponding angular displacement changes. The real-time angle of the transmission housing was determined using the calibration hole and scale in the sensor’s mechanical housing described above. A sampling point was set every 10°, with sufficient pause to stabilize the sensor output data. The results are shown in Figure 5. It can be observed that, under the same test procedure, different sensor samples exhibit minor deviations in output voltage at the same angle. These deviations are attributed to differences in rotary potentiometer resistance values and assembly errors. Additionally, the slopes and correlation coefficients for the output characteristic curves of the three tested sensor samples are very close. This verifies the performance consistency of the novel resistive angle sensor proposed in this paper for batch applications. Sensor sample 2, which has the highest correlation coefficient in the output characteristic curve, was selected for the subsequent experiments.

### 3.2. Linearity Testing

**Figure 6:**
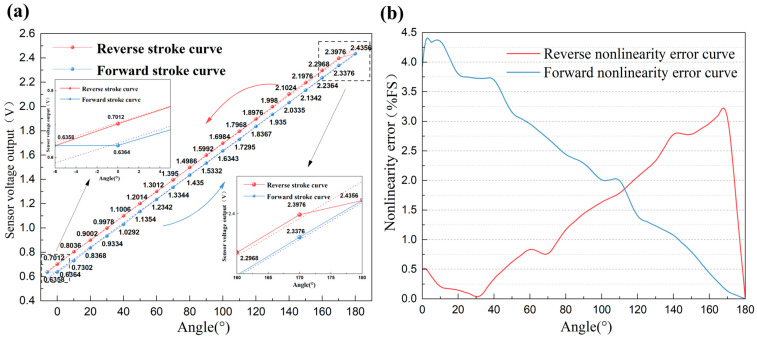
(**a**) The sensor output voltage curves for both forward and reverse strokes; (**b**) the nonlinearity error curves for both forward and reverse strokes.

The test procedure and conditions are similar to those in Section 3.1, with the following differences: The transmission housing was pushed in the opposite direction upon reaching 180° until the sensor output voltage returned to the initial value, after which the direction was reversed again. This operation was repeated five times with the sensor voltage output recorded at each sampling point during both the forward (angle increasing) and reverse (angle decreasing) strokes. Finally, the experimental data were averaged to generate the forward and reverse stroke curves, as shown in Figure 6a. The average data from 0° to 180° during both forward and reverse strokes were separately linear-fitted, and the forward-stroke equation for sensor output voltage as a function of angle can be expressed as follows:

(4) $$V_{inc}=0.01001\theta +0.63314$$

where *V_inc_* is the actual output of the sensor during the forward stroke.

Coefficient correlation:

(5) $$R_{inc}^{2}=0.99998=99.998\%$$

Similarly, the reverse-stroke equation for sensor output voltage as a function of angle can be expressed as follows:

(6) $$V_{dec}=0.00988\theta +0.70643$$

where *V_dec_* is the actual output of the sensor during the reverse stroke.

Coefficient correlation:

(7) $$R_{dec}^{2}=0.99944=99.944\%$$

It can be seen that the coefficient correlation (*R*^2^) of the fitted linear equation under both forward and reverse strokes is close to 1, so the linearity of this angle sensor is excellent. Figure 6b shows the nonlinearity error curves for both forward and reverse strokes, with a maximum percentage nonlinearity error of 4.413%.

### 3.3. Hysteresis Testing

For most sensors in practical applications, hysteresis is a significant influencing factor. The percentage hysteresis error of this angle sensor can be defined as follows:

(8) $$Hysteresis=\left|\frac{\max(V_{dec}-V_{inc})}{V_{FS}}\times 100\%\right|$$

where *V_FS_* is the sensor’s full-scale output value.

**Figure 7:**
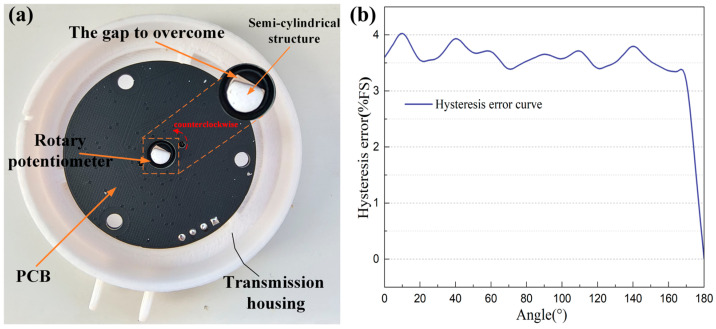
(**a**) The physical schematic diagram of the phenomenon causing hysteresis; (**b**) the percentage hysteresis error curve during forward and reverse strokes.

An analysis of the sensor’s forward and reverse stroke output voltage curves (Figure 6a) reveals that the output voltage is significantly higher during angle decrease than during increase. Moreover, only when it decreases to approximately −6°, does the output voltage become similar to the starting point (0°). This stems from unavoidable manufacturing tolerances, which hinder the perfect fit between the transmission housing’s semi-cylindrical structure and the rotary potentiometer’s central hole. Therefore, there must be a physical gap between them. When the angle starts to change (e.g., during reverse decrease from 180°), the semi-cylindrical structure must overcome this gap before altering the rotary potentiometer’s resistance. This leads to a reduction in the potentiometer’s effective angular displacement. The physical schematic diagram of the phenomenon causing hysteresis is shown in Figure 7a. As shown in Equations (4) and (6), the slopes of the two fitted linear equations for the forward and reverse strokes are approximately equal. As a result, the angle sensor’s hysteresis typically occurs at the start of an angle change, causing a shift in the forward and reverse stroke output voltages. This shift is largely proportional to the gap size from manufacturing tolerances. It can be considered that the percentage hysteresis error is nearly independent of the position where the angle change occurs. Thus, compensating for the hysteresis error in this study’s experimental results provides approximate compensation for the sensor’s overall hysteresis error. For hysteresis errors caused by mechanical coupling defects or material friction, mechanical solutions include precision machining, clearance filling, etc. Although these methods can effectively compensate for hysteresis errors in the short term, they rely on the environmental stability of filling materials and cannot eliminate time-varying disturbance factors. For example, when the sensor operates in a wide temperature range, the filling material may deform due to thermal expansion and contraction, thereby affecting the sensor’s performance [17]; periodic friction on the coupling contact surface may lead to changes in local roughness; since the gap size depends on the dimensional tolerance of the rotary potentiometer’s central hole and the transmission housing’s semi-cylindrical structure, precision machining increases the cost and production cycle time of individual sensors for large-scale mass production Therefore, it is necessary to rely on software for hysteresis error compensation to improve sensor performance. To address hysteresis in nonlinear systems, researchers have proposed differential equation-based models such as the Duhem model and Bouc–Wen model, which have compact structures but their parameters are difficult to identify. Common operator-based models include the Preisach model and Prandtl–Ishlinskii model, which describe hysteresis details more accurately but have numerous parameters and complex computations. In recent years, the Phenomenological Two-Rule model—an empirical model based on input path segmentation rules—has been widely used due to its simple and effective advantages [28]. However, this type of model requires fitting asymmetric hysteresis curves using multiple parameters such as thresholds, slopes, and hysteresis loop widths, relying on prior knowledge to adjust model parameters. Experimental data show that the hysteresis loop of the sensor caused by mechanical clearance exhibits weak nonlinearity, which is primarily induced by hysteresis phenomena. In comparison, in scenarios where weakly nonlinear hysteresis is dominated by the sensor’s mechanical coupling defects, the ANN based on the α-LMS algorithm offers significant advantages due to its higher adaptive capability and online learning ability. Figure 7b presents the percentage hysteresis error curve of this angle sensor during forward and reverse strokes, with a percentage hysteresis error of approximately 4.061%.

### 3.4. Temperature Drift and Humidity Tolerance Testing

**Figure 8:**
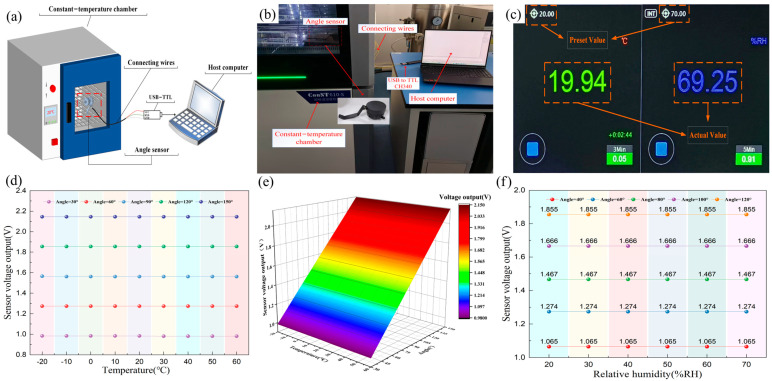
(**a**) The schematic diagram of the test platform; (**b**) the photograph of the test platform; (**c**) the GUI of the chamber (**d**) the output voltage curves of the sensor at different angles and temperatures; (**e**) the three-dimensional surface plot of the sensor output voltage as a function of both angular position and temperature; (**f**) the output voltage curves of the sensor at different angles and relative humidity.

Temperature and humidity are additional major factors contributing to sensor output drift [19], which affect resistive angle sensors more significantly than other sensor types because they may alter parameters of the components in the circuit such as the resistivity of the potentiometer. The signal conditioning circuit above has been designed to minimize temperature drift from both circuit design and component selection perspectives. This includes adding decoupling capacitors near the power pins of chips, designing a voltage follower circuit, and selecting components with extremely low temperature drift such as TPS7A05, etc. To test the impact of temperature and humidity on the sensor’s output voltage, a Const610-S precision temperature and humidity control chamber from Const was used. This chamber, with a temperature adjustment range of −30 °C to 95 °C and a humidity adjustment range of 3% RH to 95% RH, was used to conduct temperature drift and humidity tolerance tests. Then, the connecting wires between the sensor and the serial device were led out and connected to a USB-to-TTL CH340 module, which is connected to a computer for power supply. Finally, the sensor’s output voltage was acquired and visualized in real time via the host computer’s serial debugging tool. Figure 8a,b present the test platform for the novel resistive angle sensor’s temperature drift and humidity tolerance tests. Figure 8c shows the graphical user interface (GUI) of the chamber. The chamber relative humidity was maintained at 50%RH while the temperature was adjusted from −20 °C to 60 °C in 10 °C increments. At each setpoint, after temperature stabilization, the sensor was held at that temperature for one hour. Following the temperature drift tests, the chamber temperature was maintained at 20 °C, and the relative humidity was adjusted from 20%RH to 70%RH in 10%RH increments. At each humidity setpoint, after stabilization, the sensor was held at that relative humidity for one hour. Finally, the voltage outputs of the sensor at various fixed angles were recorded. The results of temperature drift test are shown in Table 1, and Figure 8d presents the output voltage curves of the sensor at different angles and temperatures. Figure 8e shows a three-dimensional surface plot of the sensor output voltage as a function of both angular position and temperature, and Figure 8f presents the output voltage curves of the sensor at different angles and relative humidity.

As the temperature rises from −20 °C to 60 °C, the sensor’s output voltage remains thermally stable with a maximum deviation of ±0.003 V. As shown in Figure 6a, with an average maximum output voltage of 2.4356 V and an average minimum output voltage of 0.6358 V over an angle range of 186°, the angle value per unit voltage (1 V) can be expressed as follows:

(9) $$\theta_{V}=\frac{\Delta \theta }{V_{\max}-V_{\min}}=\frac{186{}^{\circ}}{2.4356V-0.6538V}\approx 104.39{}^{\circ}/V$$

where *θ_V_* is the angle value per unit voltage within the sensor’s span, and Δ*θ* is the angle change during measurement. *V*_max_ and *V*_min_ are the average maximum and minimum output voltages measured in the experiment.

Thus, the angle drift caused by per unit temperature can be expressed as follows:

(10) $$\theta_{t}=\frac{\Delta V\times \theta_{V}}{\Delta T}=\frac{0.003V\times 104.39{}^{\circ}}{80\;℃}\approx 0.004{}^{\circ}$$

where *θ_t_* is the angle drift caused by per unit temperature, Δ*V* is the sensor’s output voltage drift, and Δ*T* is the temperature variation in the experiment.

As shown in Figure 8f, when the relative humidity rises from 20% RH to 70% RH, the sensor output voltage remains constant, and its humidity characteristic curve is a straight line. This is because the sensor does not use humidity-sensitive materials or electronic components, and its operating principle is independent of relative humidity. Additionally, the core component (the rotary potentiometer) is hermetically sealed and combined with the protective design of the mechanical housing, minimizing the likelihood of resistivity variations caused by environmental humidity.

Temperature compensation methods for sensors can be categorized into hardware-based and software-based approaches. Conventional hardware compensation typically employs passive and active components in circuit design, including filter networks, thermistor networks, and differential input configurations. However, these techniques enhance circuit complexity and raise system costs. In the software domain, intelligent algorithms based on machine learning have emerged as a predominant research direction for temperature compensation. Reference [29] proposed an Elman neural network—optimized based on the ant colony algorithm—to further fuse the differential output data and eliminate the temperature drift error; reference [30] proposed a temperature compensation model based on particle swarm optimization radial basis function (RBF) neural network and least squares fusion. The software compensation in the above studies requires accurate temperature data input, and the algorithms used require a large amount of data for training and optimization themselves, as well as more arithmetic resources [31]. Therefore, both hardware and software temperature compensation increase the cost of the sensor. Since the angle drift from per unit temperature (0.004°) is much smaller than this angle sensor’s span (180°), the impact of temperature on the angle sensor’s output characteristics is nearly negligible in order to be cost-effective.

### 3.5. Mechanical Vibration Testing

**Figure 9:**
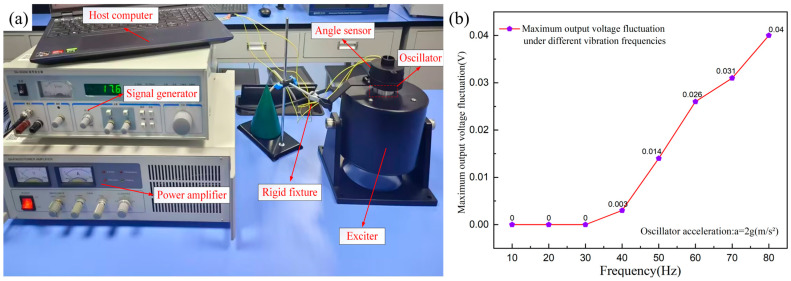
(**a**) The photograph of the test platform; (**b**) the curve of maximum output voltage fluctuation under different vibration frequencies.

The mechanical vibration test aims to simulate the mechanical vibration environment of actual industrial sites and evaluate the measurement accuracy stability and structural reliability of the angle sensor under different frequencies and amplitudes. This study conducted the mechanical vibration test on the angle sensor in accordance with the IEC 60068-2-6 standard [32]. The test platform, as shown in Figure 9a, mainly including devices such as the SA-SG030 signal generator (Wuxi Shiao, China), SA-PA020 power amplifier (Wuxi Shiao, China), and SA-JZ002 exciter (Wuxi Shiao, China). As described in Section 3.4, the influence of temperature and humidity on the sensor is almost negligible, so the experiment was conducted at room temperature. Most industrial equipment typically vibrates at frequencies from 0 to 100 Hz and with small amplitudes, so this paper focuses on investigating the effect of vibration frequency on the sensor performance. During the experiment, the sensor was horizontally placed on the oscillator of the exciter (the angle sensor is mainly used for angle measurement in the horizontal direction) and fixed with a rigid fixture to make it vibrate near the central position of the oscillator. The acceleration of the exciter oscillator was controlled at 2 g through the collaborative operation of a power amplifier and a supporting acceleration sensor. The sine wave excitation signals of different frequencies were applied to the exciter oscillator where the sensor was located using a signal generator, in order to simulate the periodic mechanical vibration of equipment in industrial environments and drive the sensor to generate expected vibrations. Before each application of the excitation signal, the transmission housing of the sensor was rotated to an appropriate position to stabilize its output voltage. Then, excitation signals with different frequencies from 0 to 80 Hz were applied at 10 Hz steps, allowing the sensor to vibrate for 1 min at each test frequency point, and the maximum voltage fluctuation of its output was observed through the host computer. After each test, the frequency was adjusted to 0 Hz, and subsequent tests were carried out according to this procedure. The results are shown in Figure 9b. As can be seen from the figure, the maximum output voltage fluctuation of the sensor shows a positive correlation with the vibration frequency. Especially at low frequencies (0–40 Hz), the sensor output voltage remains almost unchanged. However, as the vibration frequency further increases, the maximum output voltage fluctuation also increases, reaching 0.04 V at 80 Hz, corresponding to an angle deviation of approximately 4.2°. The fluctuation in the sensor’s output voltage results from mechanical vibrations in its surrounding environment, which cause the sensor’s transmission housing to vibrate. This vibration in turn leads to mechanical displacement changes in the rotary potentiometer via the semi-cylindrical structure. Therefore, the resistive angle sensor has a certain ability to resist mechanical vibration interference.

## 4. Hysteresis Compensation and Simulation Analysis

### 4.1. Adaptive Linear Neuron Model

**Figure 10:**
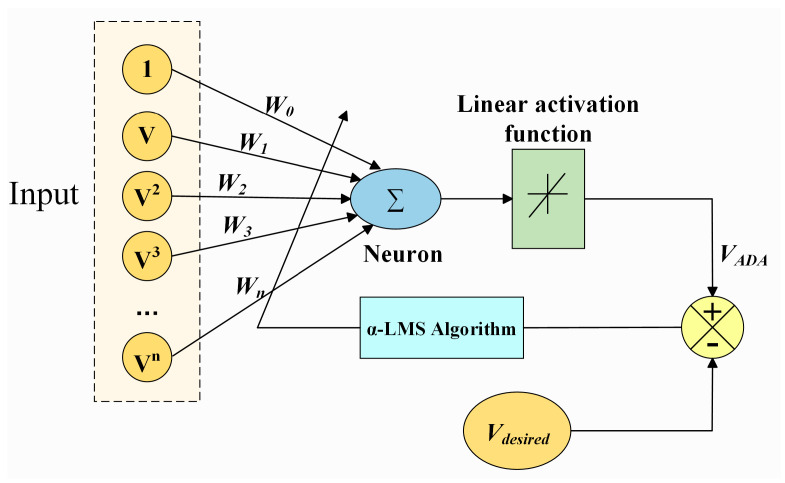
The structure of ADALINE.

As previously mentioned, the novel resistive angle sensor exhibited hysteresis error between forward and reverse measurement strokes. To address this, an ADALINE network based on the α-LMS algorithm was used in this study to linearize the sensor’s forward and reverse curves. Ideally, the two curves tend to coincide, thus minimizing the hysteresis error. The sensor output was cascaded with the ADALINE model, whose inputs were the actual sensor outputs during angle increase and decrease. Here, instead of using the sensor output V directly as a single input to the network, V was expanded into a polynomial function involving terms [1, *V*, *V*^2^, …, *V^n^*] to capture nonlinear relationships in the date and enhance the neural network’s learning ability. For an n-order model, the ADALINE model’s input is the expanded polynomial of V, with each term linked to weights [*W*_0_, *W*_1_, *W*_2_, …, *W_n_*]. The weighted sum of the inputs was computed by the neuron, and the sum result was processed through a linear activation function to ultimately output *V_ADA_*. The α-LMS algorithm compared *V_ADA_* with the desired linear output *V_desired_* and calculated the error. Based on this error, the input layer weights were updated iteratively via backpropagation, and iterative optimization proceeded until the error goal was achieved. After neural network training, *V_ADA_* approaches *V_desired_* [22,33,34]. The network’s response can be expressed by an n-order polynomial function:

(11) $$V_{ADA}=\sum_{i=0}^{k}c_{i}V^{i}$$

where *V_ADA_* is the actual output of the network, *c_i_* is the *i*th coefficient (weights of the network), *k* is the order of polynomial, and V is the actual sensor output. The structure of the adaptive linear neuron is shown in Figure 10.

### 4.2. α-LMS Algorithm

The proposed ADALINE network used the α-LMS algorithm based on the steepest descent procedure adjusting its weights and biases to minimize the mean squared error. The weight updating equation during the training of α-LMS algorithm can be expressed as follows [22]:

(12) $$W_{k+1}=W_{k}+\alpha \varepsilon_{k}\frac{X_{k}}{{\left|X_{k}\right|}^{2}}$$

where *k* is the number of iterations, *W_k_* is the weight vector of the *k*th iteration, *W*_*k*+1_ is the weight vector updated in the (*k*+1)th iteration, *α* is the learning rate parameter, and *ε_k_* is the error at the *k*th iteration.

The weight vector *W* and the normalized input vector *X* of the *n*th-order polynomial function are given in Equation (13):

(13) $$\begin{array}{l} W={\left[c_{0},c_{1},c_{2},\ldots ,c_{n}\right]}^{T}\\ X={\left[1,V,V^{2},\ldots ,V^{n}\right]}^{T} \end{array}$$

*ε_k_* in Equation (12) can be expressed as follows:

(14) $$\varepsilon^{k}=V_{k}-W_{k}^{T}X_{k}$$

where *V_k_* is the desired linear output of the sensor, *W_k_*^T^*X_k_* is the actual output of the network, and α in Equation (12) is used to adjust the weights progressively at each iterative training step. The value of α controls the stability and speed of convergence, and can be taken as any value within 0 and 1 [35,36].

### 4.3. Simulation Results and Analysis

Simulation studies have been carried out by writing the MATLAB code for the α-LMS algorithm. The linear approximation equation for the sensor was obtained by connecting the two extreme points of the output voltage curves under both forward and reverse strokes and can be expressed as V_desired_ = 0.00968θ + 0.69386. The data for network training were obtained from Figure 6a. The polynomial order of the network output is empirically determined, typically being second-order or third-order. To simplify subsequent hardware implementation and reduce complexity, this study employs a second-order polynomial. The entire dataset was randomly shuffled and partitioned into training and test subsets in an 8:2 ratio. The training dataset was utilized to train the neural network through the iterative adjustment of model parameters (weights), while the test dataset served to evaluate the model’s generalization capability on unseen data. To enhance the sample size of the neural network model, this paper employs interpolation functions for preprocessing the input data, thereby increasing the number of samples from the original 40 to 374. The initial weights of the network were randomly generated within the range of 0–1. For fixed iteration counts and learning rate parameters, the network was trained with the input data, and its root mean square error was determined. Input and output data points were normalized by dividing the input and output vectors by their respective maximum values. The percentage root mean square error (RMSE) of the network can be expressed as follows:

(15) $$rmse=\left(\sqrt{\frac{1}{N}\sum_{i=1}^{N}\left(V_{desired}-V_{ADA}\right)}\right)\times 100\%$$

where *rmse* is the percentage root mean square error, *V_desired_* is the normalized desired approximate linear output of the ADALINE network, *V_ADA_* is the normalized actual output of the network, and *N* is the total number of data points.

The network was trained until the RMSE converged to a minimum. Finally, the normalized output data were denormalized to obtain the sensor output voltage values for forward and reverse strokes within the angular range of 0–180°. The number of iterations and learning rate parameter α were continuously adjusted until the RMSE reached a relative minimum. The adaptive linear neuron was ultimately trained with a learning rate parameter of 0.05 and 50,000 iterations. Thus, the *rmse* of the reverse stroke curve can be obtained as 0.261% with the binomial equation expressed as Equation (16):

(16) $$y=-2\times 10^{-7}x^{2}+0.0097x+0.6938$$

The *rmse* of the forward stroke curve can be obtained as 0.367% with the binomial equation expressed as Equation (17):

(17) $$y=-4\times 10^{-7}x^{2}+0.0098x+0.6868$$

The mean squared error (MSE) is commonly used to evaluate the generalization capability of neural networks, which quantifies the discrepancy between predicted and true values. A smaller MSE indicates better generalization performance of the model. The specific formula is as follows:

(18) $$MSE=\frac{1}{n}{\sum_{i=1}^{n}\left(V_{i}-\hat{V_{i}}\right)}^{2}$$

where *n* is the number of samples in the dataset, *V_i_* is the true voltage value of the *i*th sample, and $\hat{V_{i}}$ is the predicted voltage value of the *i*th sample.

**Figure 11:**
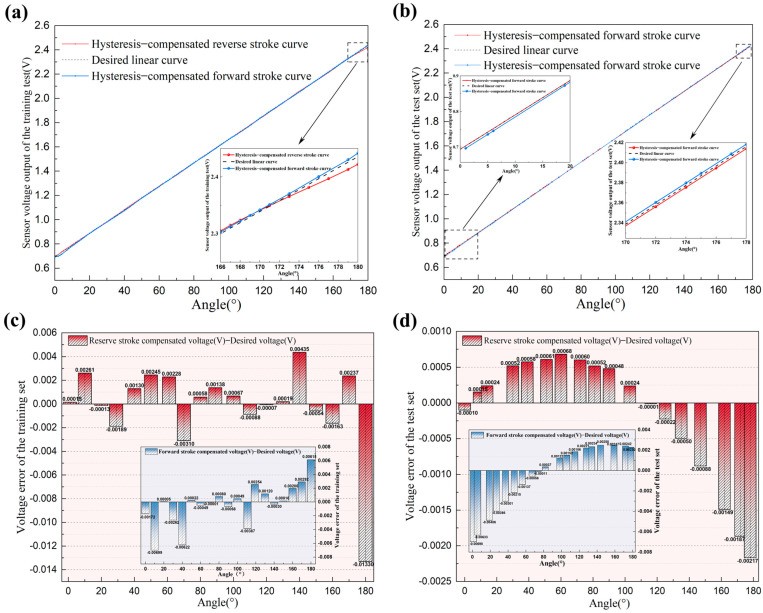
(**a**) The output voltage curves of the training set under forward and reverse strokes; (**b**) the output voltage curves of the test set under forward and reverse strokes; (**c**) the errors between the model-predicted voltages and the desired linear voltages of the training set; (**d**) the errors between the model-predicted voltages and the desired linear voltages of the test set.

**Figure 12:**
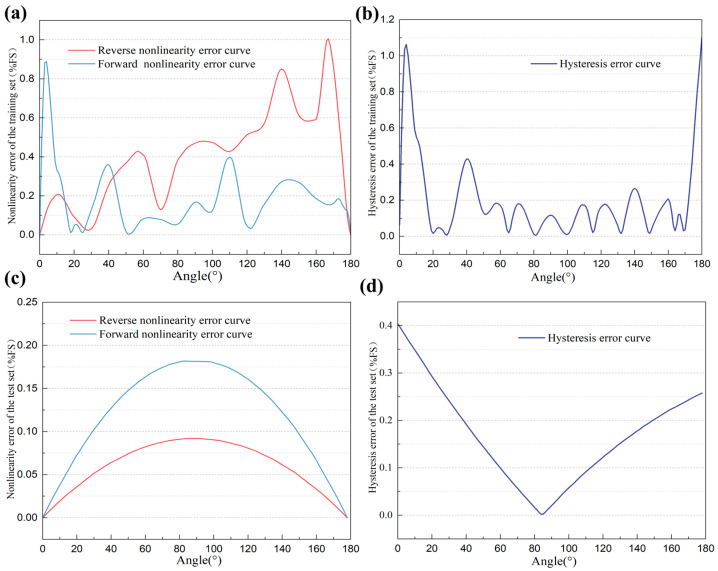
(**a**) The percentage nonlinearity error curves of the training set; (**b**) the percentage hysteresis error curves of the training set; (**c**) the percentage nonlinearity error curves of the test set; (**d**) the percentage hysteresis error curves of the test set.

The results indicate that the MSE of the forward stroke in the test set is approximately 0.00121, and that of the reverse stroke is approximately 0.00106. For the training set, the forward stroke MSE is approximately 0.00116, and the reverse stroke MSE is approximately 0.00136. These data reveal that both the test and training sets exhibit very low MSE values for both stroke directions, and the MSEs under the same stroke conditions are remarkably close. This demonstrates that the neural network model developed in this study possesses excellent generalization capability without overfitting. Due to the random shuffling of the training data, which may cause incomplete overlap with the original input values, this paper employs interpolation alignment processing to compare the outputs at the same input values before and after hysteresis compensation. The comparison results of the sensor’s partial output voltages under forward and reverse strokes before and after hysteresis compensation are presented in Table 2. The sensor’s partial output voltages under forward and reverse strokes before and after hysteresis compensation. Figure 11a,b show the output voltage curves of the training set and test set under forward and reverse strokes after hysteresis compensation, respectively. To visually display the errors between the model-predicted voltages and the desired linear voltages before and after hysteresis compensation, bar charts are plotted as shown in Figure 11c,d. Figure 12a,b present the percentage nonlinearity error curves and percentage hysteresis error curves of the training set. Figure 12c,d show the percentage nonlinearity error curves and percentage hysteresis error curves of the test set.

From the above table and figures, it is evident that the forward and reverse stroke curves of the sensor’s output voltage nearly perfectly overlap after hysteresis compensation. The percentage nonlinearity error of the training set is reduced from the original 4.413% to 1.006% and the percentage hysteresis error is reduced from the original 4.061% to 1.105%. The results of the test set reflect the model’s performance in practical applications, exhibiting a percentage nonlinearity error of 0.182% and a percentage hysteresis error of 0.404%. These results indicate that the ADALINE network based on the α-LMS algorithm effectively compensates nonlinearity and hysteresis in the novel resistive angle sensor, improving measurement accuracy. Moreover, this method is simple and easily implementable on embedded hardware. A further reduction in hysteresis error can be achieved by increasing the number of training data points. However, this may prolong the calibration time of the sensor’s hardware compensation circuit, and further research is needed to prove this.

## 5. Discussion

As described in Section 3.1, the different sensors of the same batch exhibit slight deviations in output voltage at the same angle. These deviations, which are random in nature, are attributed to differences in rotary potentiometer resistance values and assembly errors. Therefore, each sensor requires individual calibration and training of the ADALINE model to ensure maximum measurement accuracy. After long-term operation, the angle sensor may exhibit reduced linearity due to wear and aging of the rotary potentiometer. The algorithm is a core designed to compensate for hysteresis errors caused by inherent mechanical clearances, but it can also address nonlinear errors. Therefore, after prolonged sensor usage, parameter fine-tuning (e.g., updating weights based on minimal calibration data) can replace complete neural network retraining to ensure long-term algorithmic robustness. Limited by the sample size, no systematic experiments were conducted across batches of sensors in this paper. Future work will incorporate data from multiple batches of similar sensors to quantify the transferability boundaries of the model.

The proposed sensor has been compared with the prior art in terms of their performance parameter and output characteristics, as shown in Table 3. It is obvious that this resistive angle sensor has a great advantage in cost-sensitive applications where high accuracy is not required compared with the prior art and commercial sensors. It should be noted that, although the performance and resolution of the sensor can be improved by selecting a microcontroller with higher ADC bits or a higher-precision compensation algorithm, this incurs additional costs.

## 6. Conclusions

This paper presents a novel resistive angle sensor based on a rotary potentiometer. This sensor is low cost, has high temperature stability, and has strong environmental adaptability, meeting the low-cost angle measurement needs in industrial automation and EMI-sensitive applications. The measurement accuracy of the sensor has been optimized from both hardware and software perspectives. On the one hand, the hardware circuit design based on the principle of ADC endows the sensor with excellent output linearity, high temperature stability, and strong EMI immunity. On the other hand, to address nonlinearity and hysteresis errors caused by wear, aging, and manufacturing tolerances in resistive angle sensors, this study employs an adaptive linear neuron based on the α-LMS algorithm for compensation. Consequently, the sensor’s overall nonlinearity error is reduced from 4.413% to 0.182%, and the overall hysteresis error is reduced from 4.061% to 0.404%. The contributions of this study offer valuable insights for the development of high-precision resistive angle sensors. Future work will focus on implementing the proposed algorithm in embedded hardware and conducting long-term stability tests to further optimize sensor performance and expand its applications.

## Figures and Tables

**Table 1:** The voltage output of the sensor (V) under varying angles and temperatures.

Angle (°)	Test Temperature (°C)								
	−20	−10	0	10	20	30	40	50	60
30°	0.984	0.984	0.984	0.984	0.984	0.981	0.981	0.981	0.981
60°	1.274	1.274	1.274	1.274	1.274	1.274	1.274	1.274	1.274
90°	1.565	1.562	1.562	1.562	1.562	1.562	1.562	1.562	1.562
120°	1.855	1.855	1.855	1.855	1.855	1.855	1.855	1.855	1.855
150°	2.146	2.146	2.146	2.146	2.146	2.146	2.146	2.146	2.146

**Table 2:** The sensor’s partial output voltages under forward and reverse strokes before and after hysteresis compensation.

Angle (°)	Pre-Compensation Forward Stroke Voltage (V)	Post-Compensation Forward Stroke Voltage (V)	Pre-Compensation Reverse Stroke Voltage (V)	Post-Compensation Reverse Stroke Voltage (V)
0	0.6364	0.6922	0.7012	0.6936
20	0.8368	0.8875	0.9002	0.8873
40	1.0292	1.0747	1.1006	1.0822
60	1.2342	1.2739	1.3012	1.2769
80	1.4350	1.4688	1.4986	1.4686
100	1.6343	1.6620	1.6984	1.6622
120	1.8367	1.8577	1.8976	1.8549
140	2.0335	2.0482	2.1024	2.0529
160	2.2364	2.2441	2.2968	2.2405
180	2.4356	2.4418	2.4356	2.4223

**Table 3:** The comparison of this work with the prior art and commercially available products.

Parameters	Resistive Sensors		Inductive Sensors		Magnetoresistive Sensors		Optical Sensors	
	This Work	WDD35D4 from MIRAN, CHN *	[37]	RI360P1 from TURCK, GER *	[6]	ADA4571 from ADI, USA *	[8]	E6CP-A from Omron, CHN *
Range	180°	345°	360°	360°	360°	180°	360°	360°
Resolution	0.31°	0.07°	0.08°	0.09°	0.1°	0.5°	0.02°	8 bit
Output characteristics	Linear	Linear	Linear	Linear	Linear	Non-Linear	Linear	Non-Linear
Nonlinearity error of full scale	0.18%	0.3%	0.25%	0.3%	-	±0.7°	1.15%	±1°
temperature drift (per °C)	0.004°	0.003°	-	≤0.036°	-	≤0.004°	-	-
Manufacturing complexity	Simple	Moderate	Simple	Moderate	Moderate	Complex	Complex	Complex
Sensitive to EMI	No	No	No	Yes	Yes	Yes	No	No
Cost	Low	Moderate	Low	Moderate	Moderate	Low	High	Moderate

* Commercially available products.

## Data Availability

Data are contained within the article.
